# Lower Serum Creatinine Is Associated with Low Bone Mineral Density in Subjects without Overt Nephropathy

**DOI:** 10.1371/journal.pone.0133062

**Published:** 2015-07-24

**Authors:** Ji Hye Huh, Soo In Choi, Jung Soo Lim, Choon Hee Chung, Jang Yel Shin, Mi Young Lee

**Affiliations:** Department of Internal Medicine, Wonju College of Medicine, Yonsei University, Ilsan-Dong, Wonju-Si, Gangwon-Do, Korea; Universidade de São Paulo, BRAZIL

## Abstract

**Background:**

Low skeletal muscle mass is associated with deterioration of bone mineral density. Because serum creatinine can serve as a marker of muscle mass, we evaluated the relationship between serum creatinine and bone mineral density in an older population with normal renal function.

**Methods:**

Data from a total of 8,648 participants (4,573 men and 4,075 postmenopausal women) aged 45–95 years with an estimated glomerular filtration rate >60 ml/min/1.73 m2 were analyzed from the Fourth Korea National Health and Nutrition Examination Survey (2008–2010). Bone mineral density (BMD) and appendicular muscle mass (ASM) were measured using dual-energy X-ray absorptiometry. Receiver operating characteristic curve analysis revealed that the cut points of serum creatinine for sarcopenia were below 0.88 mg/dl in men and 0.75 mg/dl in women. Subjects were divided into two groups: low creatinine and upper normal creatinine according to the cut point value of serum creatinine for sarcopenia.

**Results:**

In partial correlation analysis adjusted for age, serum creatinine was positively associated with both BMD and ASM. Subjects with low serum creatinine were at a higher risk for low BMD (T-score ≤ –1.0) at the femur neck, total hip and lumbar spine in men, and at the total hip and lumbar spine in women after adjustment for confounding factors. Each standard deviation increase in serum creatinine was significantly associated with reduction in the likelihood of low BMD at the total hip and lumbar spine in both sexes (men: odds ratio (OR) = 0.84 [95% CI = 0.74−0.96] at the total hip, OR = 0.8 [95% CI = 0.68−0.96] at the lumbar spine; women: OR = 0.83 [95% CI = 0.73–0.95] at the total hip, OR=0.81 [95% CI = 0.67–0.99] at the lumbar spine).

**Conclusions:**

Serum creatinine reflected muscle mass, and low serum creatinine was independently associated with low bone mineral density in subjects with normal kidney function.

## Introduction

Growing evidence supports cross-talk between bone and muscle because they have common genetic, nutritional, lifestyle, and hormonal determinants [1]. Interactions between muscle and bone can affect bone strength [2], and it has been previously documented that bone functions as a musculoskeletal unit and adapts to the mechanical loads exerted by skeletal muscle [3]. In addition, a progressive decline in bone mineral density (BMD), muscle mass and muscle strength have common key features of the aging process. Accordingly, sarcopenia, the age-related loss of muscle mass, has been suggested as a major risk factor for low BMD and fracture in several epidemiological studies [4–6]. Therefore, identification of sarcopenia is an important factor in older populations suggesting whether individuals have decreased BMD and are therefore at high risk of fragility fracture. Although dual-energy X-ray absorptiometry (DXA) is currently accepted as the gold standard method to measure both muscle mass and BMD, it is expensive and not easily accessible for many populations.

Serum creatinine is primarily a metabolite of creatine phosphate, almost all of which is found in skeletal muscle. Because the amount of creatinine per unit of skeletal muscle mass and the breakdown rate of creatine are both consistent, plasma creatinine concentration is a stable, direct reflection of skeletal muscle mass [7]. In addition, because 24-h urinary creatinine excretion is highly correlated with muscle mass estimates determined using DXA [8], and serum creatinine is highly correlated with 24-h urine excretion in subjects with normal renal function [9], serum creatinine could represent an acceptable and easily measured surrogate marker of muscle mass. Considering that skeletal muscle is a major target tissue of insulin [10], several previous studies have reported lower serum creatinine (reflecting lower skeletal muscle) to be associated with metabolic disorders such as insulin resistance and type 2 diabetes [11, 12]. From those findings and considering the bone-muscle relationship, we speculated that lower serum creatinine might also be associated with deterioration of BMD, especially in subjects without renal insufficiency. However, few studies have reported the association between serum creatinine and BMD.

Therefore, the aim of the present study was to investigate the associations between serum creatinine and BMD in adults with normal kidney function using data from the general Korean population. We also examined whether those associations differ by sex or skeletal sites. We hypothesized that lower serum creatinine, reflecting low muscle mass, might be related to decreased BMD and that serum creatinine could provide information about an individual’s bone health as well as muscle health in subjects without renal insufficiency.

## Materials and Methods

### Study population and design

The Korea National Health and Nutrition Examination Survey (KNHANES) has been performed periodically since 1998 by the Division of Chronic Disease Surveillance of the Korean Centers for Disease Control and Prevention to assess the health and nutritional status of the civilian, non-institutionalized population of Korea. The KNHANES IV, V was a cross-sectional and nationally representative survey conducted from 2008 to 2010 which are available on the KNHANES website (https://knhanes.cdc.go.kr/knhanes/sub03/sub03_02_02.do; S1 Dataset). The survey was composed of a health interview survey, a nutrition survey, and a health examination survey. The data were collected by household interviews and by direct, standardized physical examinations conducted in mobile examination centers. Nutritional status and medical history were evaluated using a 24-h recall method. Regular exercise was indicated as “yes” when the subject exercised for more than 20 min at a time more than three times per week. Subjects with any pathological disorders (such as cancer, hyperthyroidism, malabsorption, or hepatic failure) or subjects using medications (such as corticosteroids, heparin, or anticonvulsants) known to alter calcium and bone metabolism were excluded from our analysis. We also excluded subjects who used testosterone, anabolic steroids or antiresorptive agents and who have definite renal insufficiency (estimated glomerular filtration rate <60 ml/min/1.73 m^2^). Among those who participated in the survey and met our inclusion criteria, 8,648 participants were 45 years or older (4,573 men and 4,075 postmenopausal women).

### Ethics statement

Because the KNHANES IV survey data are publicly available, ethical approval was not required for this study. Prior to the survey, all participants were informed that they had been randomly chosen to participate in the KNHANES IV survey with the right to refuse to be involved in further analyses, and signed informed consents were obtained. The data we used from the KNHANES database were fully anonymized.

### Measurements and definitions of sarcopenia

Total body fat and appendicular skeletal muscle mass (ASM) as well as BMD at the lumbar spine (LS) (L1–4) and hip region were measured using DXA (QDR 4500A; Hologic Inc., Waltham, MA). All men and non-pregnant women aged 20 years and older who received a physical examination in the mobile centers were eligible for bone densitometry analysis unless they had previously fractured both hips. Low BMD was defined as a T-score of –1.0 or less. Relative ASM was calculated as the sum of the mass of skeletal muscle in the arms and legs, divided by the square of the height (ASM/ht^2^ in kg/m^2^). A subject was classified as having sarcopenia when he or she had a relative ASM less than one standard deviation (SD) below the sex-specific normal mean for the young reference group (healthy men and women aged 20–39 years) [13]. The cutoff point for sarcopenia was 7.86 kg/m^2^ for men and 5.71 kg/m^2^ for women.

### Biochemical analysis

Collected blood samples were immediately refrigerated, transported to the Central Testing Institute in Seoul, Korea, and analyzed within 24 h. Serum creatinine levels were determined using a Hitachi 7600 automated chemistry analyzer (Hitachi, Tokyo, Japan) with Creatinine plus (Roche Diagnostics). The estimated glomerular filtration rate derived from the Chronic Kidney Disease Epidemiology Collaboration equation was used to assess renal function; this method is more accurate than previous indices, such as the Modification of Diet in Renal Disease Study equation [14]. Fasting plasma glucose and cholesterol levels were measured with a Hitachi 700–110 chemistry analyzer (Hitachi). Serum 25-hydroxyvitamin D [25(OH)D] concentrations were measured by radioimmunoassay (DiaSorin Inc., Stillwater, MN, USA) using a γ-counter (1470 Wizard; PerkinElmer, Turku, Finland). A chemiluminescence immunoassay (N-tact PTH assay; DiaSorin) was used to measure serum intact parathyroid hormone. Fasting insulin (INS-IRMA; Biosource, Nivelles, Belgium) was measured by an immunoradiometric assay. The homeostasis model assessment of insulin resistance (HOMA-IR) was calculated using the following formula: fasting (plasma glucose (mg/dL) x fasting insulin (mIU/mL))/22.5 [15].

### Statistical analysis

Statistical analyses were conducted using IBM’s SPSS version 20.0 for Windows (IBM Corp., Armonk, NY, USA). A receiver operating characteristic curve (ROC) was created to find the cut-off point of serum creatinine for predicting sarcopenia. The optimal cut point was 0.88 in men (sensitivity was 57%; specificity was 57% at this level) and 0.75 in women (sensitivity was 70%; specificity was 40% at this level). We classified subjects into two groups according to this cut-off point value of serum creatinine. An independent, two-sample *t*-test was used to compare differences in the mean values of baseline parameters among the groups. For categorical variables, a chi-square test was used to compare the frequencies among the groups. Partial correlation analyses were used to evaluate the association between serum creatinine and body composition indices with adjustments made for age. Multiple linear regression analysis was then used to determine the association between serum creatinine and the outcome (total hip, femoral neck and lumbar spine BMD) adjusted for confounding factors. Multiple logistic regression analysis was used to examine the association between sarcopenia and low BMD, with the results expressed as odds ratios (OR) and 95% confidence intervals (CI).

## Results

### Clinical characteristics of the study population

The demographic and clinical characteristics of the patients, who were classified into two groups according to sex-specific cutoff values of creatinine for sarcopenia (Men: serum creatinine ≤ 0.8 mg/dL, Women: ≤ 0.7 mg/dL), are shown in Table 1. The mean age was 60.16±10.06 years (range, 45–93 years) in men and 62.96±9.07 years (range, 45–95 years) in postmenopausal women. The low creatinine group had lower body mass index, weight, waist circumference and ASM in both sexes. BMD at the total hip (TH), femoral neck (FN), and lumbar spine (LS) was significantly lower in the low creatinine group than in the upper normal creatinine group in men, but only BMD at LS was significantly lower in the low creatinine group in postmenopausal women. Fasting insulin and HOMA-IR were higher in the upper normal creatinine group than in the low creatinine group. The frequency of alcohol consumption and the proportion of current smokers were both higher in the low creatinine group in both sexes.

**Table 1:** Clinical characteristics of the subjects.

Variable (unit)	Men			Women		
	Low creatinine (n = 1,189)	Upper normal creatinine (n = 3,384)	*P*- value	Low creatinine (n = 2,845)	Upper normal creatinine (n = 1,230)	*P*- value
Serum creatinine (range, mg/dL)	0.78±0.06 (0.5–0.8)	1.01±0.09 (0.9–1.3)	<0.001	0.65±0.06(0.4–0.7)	0.83±0.05(0.8–1.0)	<0.001
Age (yr)	61.1 ± 9.71	59.83 ± 10.17	<0.001	62.39 ± 8.87	64.27 ± 9.41	<0.001
Anthropometry						
Height (cm)	166.16 ± 6.31	167.3 ± 5.99	<0.001	153.16 ± 5.81	153.54 ± 5.92	0.054
Weight (kg)	63.71 ± 9.97	67.48 ± 9.75	<0.001	56.54 ± 8.57	57.65 ± 8.68	<0.001
BMI (kg/m^2^)	23.02± 2.47	24.57 ± 2.42	<0.001	20.51 ± 2.54	24.25 ± 3.22	<0.001
Waist circumference (cm)	83.64 ± 8.85	85.71 ± 8.46	<0.001	82.07 ± 9.27	82.92 ± 9.25	<0.001
Appendicular skeletal muscle (kg)	20.2 ± 3	21.33 ± 3.06	<0.001	13.94 ± 1.94	14.28 ± 2.08	<0.001
RASM^a^ (kg/m^2^)	7.29 ± 085	7.58 ± 0.87	<0.001	5.93 ± 0.68	6.03 ± 0.71	<0.001
Body fat (%)	21.12 ± 5.34	22.57 ± 4.95	0.308	33.98 ± 5.51	34.41 ± 5.24	0.023
Bone mineral density (g/cm^2^)						
Total hip	0.91 ± 0.13	0.944 ± 0.13	<0.001	0.78 ± 0.12	0.77 ± 0.13	0.005
Femoral neck	0.74 ± 0.12	0.76 ± 0.12	<0.001	0.63 ± 0.11	0.61 ± 0.11	<0.001
Lumbar spine	0.91 ± 0.15	0.96 ± 0.15	<0.001	0.8 ± 0.14	0.81 ± 0.14	0.013
Hormones and biochemistry						
25(OH)D (ng/mL)	22.27 ± 7.78	21.12 ± 7.2	<0.001	19.1 ± 7.19	19.36 ± 7.36	0.299
PTH (pg/mL)	62.99 ± 22.3	65.88 ± 25.76	0.002	67.4 ± 32.07	67.93 ± 27.93	0.627
Fasting glucose (mg/dL)	106.22 ± 30.43	103.9 ± 27.04	0.02	100.32 ± 22.86	101.95 ± 24.89	0.05
Fasting insulin (mIU/mL)	8.82 ± 4.44	9.78 ± 5.69	<0.001	10.02 ± 6.78	10.86 ± 5.87	<0.001
HOMA-IR	2.37 ± 1.65	2.57 ± 2.24	0.004	2.58 ± 3.27	2.82 ± 1.98	0.02
eGFR (mL/min/1.73 m^2^)	109.33 ± 11.21	81.64 ± 9.28	<0.001	99.64 ± 12.9	74.4 ± 5.67	<0.001
eGFR			<0.001			<0.001
60–70 mL/min/1.73 m^2^ (%)	0 (0%)	550 (16.3%)		0 (0%)	304 (24.8%)	
71–80 mL/min/1.73 m^2^ (%)	0 (0%)	998 (29.5%)		181 (6.3%)	725 (58.9%)	
81–90 mL/min/1.73 m^2^ (%)	4 (0.3%)	1147 (33.9%)		707 (24.9%)	182 (14.8%)	
≥ 91 mL/min/1.73 m^2^ (%)	1185 (99.7%)	689 (20.3%)		1957 (68.8%)	19 (1.5%)	
Daily calcium intake (mg/d)	536.36±343.52	542.24±358.89	0.641	424.75±306.59	395.56±457.68	0.017
Alcohol intake ≥ one time/week (%)	855 (72.6)	2405 (67.3)	0.001	752 (26.6)	302 (21.3)	<0.001
Sarcopenia (%)	816 (75.9)	1792 (64.5)	<0.001	1046 (38.2)	469 (34.3)	0.016
Regular exercise^b^ (%)	216 (18.3)	655 (18.3)	0.994	354 (12.5)	188 (13.2)	0.511
Current smoker (%)	495 (42)	186 (33.0)	<0.001	495 (42)	1186 (33.1)	<0.001
Estrogen replacement therapy (%)	-	-	-	445 (16.5)	215 (15.8)	0.56

Data presented as n (%) or mean ± standard deviation. BMI, body mass index; RASM, relative appendicular skeletal muscle mass; PTH, parathyroid hormone; HOMA-IR, homeostasis model assessment of insulin resistance; eGFR, estimated glomerular filtration rate

^a^Appendicular skeletal muscle mass divided by height squared

^b^Regular exercise indicated that the subject performed vigorous exercise for more than 20 min at a time more than three times per week

### Relationships between serum creatinine, muscle mass and bone density

As shown in S1 Table, we found a positive correlation between serum creatinine and BMD at TH, FN, and LS after adjusting for age (R = 0.162, *P* < 0.001 at TH; R = 0.221, *P* < 0.001 at FN; R = 0.274, *P* < 0.001 at LS). We also found a positive correlation between serum creatinine and total skeletal muscle mass (R = 0.424, *P* < 0.001), ASM (R = 0.43, *P* < 0.001) and relative ASM (R = 0.362, *P* < 0.001). Serum creatinine was also negatively associated with body fat (%) (R = -0.309, *P* < 0.001). Table 2 presents the independent contribution of serum creatinine to BMD at each site using multiple linear regression analysis. Even after adjustment for all potential confounders, an increase in serum creatinine significantly contributed to an increase of BMD at TH, FN, and LS in both sexes. The association between serum creatinine and BMD was more prominent in men than in women.

**Table 2:** Multivariate linear regression analysis for bone mineral density in men and women.

Variables	**Total hip**			**Femoral neck**			**Lumbar spine**		
	**β** ^**a**^	**SE**	*P*-value	**β** ^**a**^	**SE**	*P*-value	**β** ^**a**^	**SE**	*P*-value
*Men*									
Serum creatinine (mg/dL)	0.752	0.115	<0.001	0.503	0.12	<0.001	1.135	0.169	<0.001
Variables	**Total hip**			**Femoral neck**			**Lumbar spine**		
	**β** ^b^	**SE**	*P*-value	**β** ^b^	**SE**	*P*-value	**β** ^b^	**SE**	*P*-value
*Women*									
Serum creatinine (mg/dL)	0.494	0.141	<0.001	0.323	0.136	0.018	1.102	0.181	<0.001

^a^Results expressed as β coefficients. Data adjusted for age, body fat (%), HOMA-IR, current smoking status, alcohol intake, regular exercise, vitamin D and daily calcium intake (mg/day)

^b^Results expressed as β coefficients. Data adjusted for age, body fat (%), HOMA-IR, current smoking status, alcohol intake, regular exercise, vitamin D and daily calcium intake (mg/day) and estrogen replacement therapy.

### Sex-specific prevalence of low BMD according to serum creatinine levels

**Fig 1:**
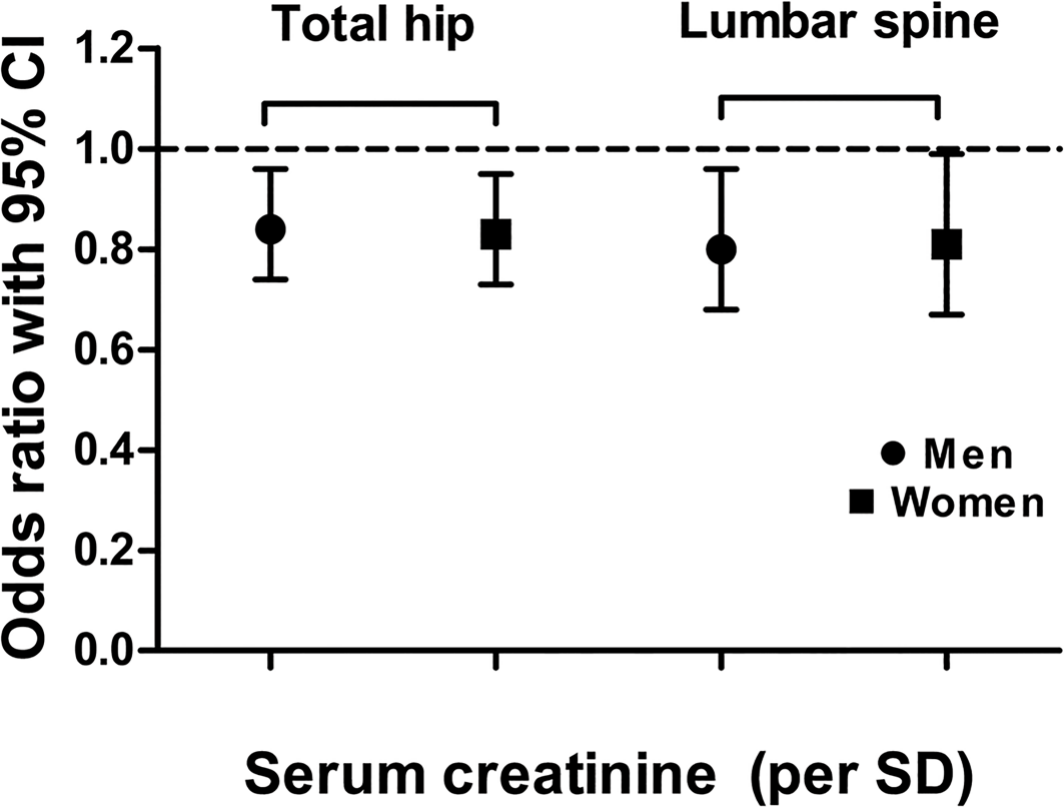
Adjusted odds ratios with 95% confidence interval for the presence of low bone mineral density for each standard deviation (SD) increase in serum creatinine. *Data were adjusted for age, current smoking status, regular exercise, daily calcium intake (mg/d), HOMA-IR, vitamin D, body fat (%) and estrogen replacement therapy (in women).

After adjusting for age, the low creatinine group had a significantly elevated OR for low BMD at all sites in both sexes (Table 3). After further adjusting for other conventional confounding covariates (Models 2 and 3), the low creatinine group of men still had a significantly elevated OR for low BMD at all sites, but the low creatinine group of postmenopausal women had an elevated OR for low BMD at only TH and LS. Each standard deviation increase in serum creatinine was significantly associated with a 16% reduction in the likelihood of low BMD at TH and a 20% at LS in men (OR = 0.84, 95% CI = 0.74−0.96 at TH; OR = 0.8, 95% CI = 0.68−0.96 at LS) (Fig 1). In women, each standard deviation increase in serum creatinine was significantly associated with a 17% reduction in the likelihood of low BMD at TH and a 19% reduction at LS (OR = 0.83, 95% CI = 0.73−0.95 at TH, OR = 0.81, 95% CI = 0.67−0.99 at LS). However, each standard deviation increase in serum creatinine was not significantly associated with a low BMD at FN in both sexes (OR = 0.83, 95% CI = 0.69–1 in men and OR = 0.85, 95% CI = 0.68–1 in women).

**Table 3:** Multivariate odds ratio and 95% confidence interval for low bone mineral density^a^ according to serum creatinine.

	Men			Women		
	Upper normal creatinine	Low creatinine	*P-value*	Upper normal creatinine	Low creatinine	*P-value*
Total hip						
Incidence^b^ (%)	392 (13.7%)	194 (17.6%)	0.002	566 (39.8%)	995 (35.2%)	0.004
Model 1	reference	1.32 (1.08,1.61)	0.008	reference	1.20(1.02,1.41)	0.028
Model 2	reference	1.32 (1.08,1.62)	0.005	reference	1.23(1.04,1.46)	0.014
Model 3	reference	1.26 (1.01,1.57)	0.043	reference	1.20 (1.01,1.43)	0.038
Femur neck						
Incidence^b^ (%)	1081 (37.7%)	501 (45.5%)	<0.001	1113 (78.3%)	2115(74.9%)	0.016
Model 1	reference	1.35 (1.16,1.56)	<0.001	reference	1.20 (1.01,1.43)	0.039
Model 2	reference	1.33 (1.14,1.55)	<0.001	reference	1.17 (0.98,1.41)	0.089
Model 3	reference	1.28 (1.08,1.52)	0.004	reference	1.11 (0.92,1.34)	0.27
Lumbar spine						
Incidence^b^ (%)	1050 (37.3%)	516 (48.1%)	**<0.001**	1011(73%)	2066 (74.9%)	0.19
Model 1	reference	1.48 (1.28,1.71)	**<0.001**	reference	1.44 (1.22,1.69)	<0.001
Model 2	reference	1.48 (1.27,1.71)	<0.001	reference	1.39 (1.17,1.65)	<0.001
Model 3	reference	1.38 (1.16,1.65)	<0.001	reference	1.36 (1.14,1.62)	0.001

^a^Low bone mineral density: T-score ≤ –1.0

^b^Data are presented using a Chi-square test

Model 1: adjusted for age; Model 2: Model 1+ further adjusted for regular exercise, alcohol intake, current smoking status, 25(OH)D and estrogen replacement therapy (women); Model 3: Model 2+ further adjusted for HOMA-IR, daily calcium intake and body fat (%).

## Discussion

In this general population-based study of subjects with normal renal function, we found that serum creatinine was closely associated with ASM. Additionally, we demonstrated that significantly decreased BMD was observed in subjects with low serum creatinine (≤ 0.8 mg/dL in men and ≤ 0.7 mg/dL in postmenopausal women). The association between serum creatinine and BMD was more prominent in men than in women. These discoveries provide the first clinical evidence for the notion that subjects with extremely low serum creatinine are at high risk for reduced BMD.

Reduced muscle mass, or sarcopenia, is a well-known risk factor for osteoporosis. Reduced muscle mass affects balance and thereby increases the risk of falls and subsequent fractures [16]. In this way, gradual age-related decline in bone and muscle (i.e., osteoporosis and sarcopenia) can result in increased morbidity and mortality [17]. Considering the close relationship between sarcopenia and osteoporosis and the effects of muscle mass on fracture risk, identification and treatment of those conditions is important in older populations. Therefore, recent studies have suggested a more inclusive name be given to the combination of sarcopenia and osteoporosis, such as ‘dysmobility syndrome,’ which integrates their pathogenesis and unites them as a single therapeutic target [18]. However, although osteoporosis has been clearly defined, the definition of sarcopenia remains unclear [19]. In addition, although DXA is the currently accepted gold standard test for evaluating body composition, including both bone density and muscle mass [20], it is not easily accessible or commonly available to general populations because of time and cost. From this background, we hypothesized that serum creatinine, known to be a stable marker of skeletal muscle mass [21], could be related to bone health status.

Several studies have reported correlations between serum creatinine and lean body mass, estimated anthropometrically using bioimpedence analysis or DXA [22, 23]. Consistent with these findings, we observed that serum creatinine was significantly associated with total and appendicular skeletal muscle mass. Therefore, we further calculated the cut point of serum creatinine for the presence of sarcopenia and it was ≤ 0.8 mg/dL in men and ≤ 0.7 mg/dL in postmenopausal women. Similarly, Harita et al. demonstrated that those who had serum creatinine levels ≤0.6 mg/dl was associated with increased the risk of type 2 diabetes [11]. However, they did not assess the risk of lower creatinine for diabetes considering gender differences although skeletal muscle mass might differ between women and men. Taken together, we can speculate that serum creatinine, a cheap and simple method, can be used as a marker to assess individual’s skeletal health status and it can be also alternative for body composition analysis in subjects with normal renal function.

Chronic kidney disease, especially stage 3 or over, is a well-known risk factor for low BMD [24] and sarcopenia [25]. Our study, which excluded subjects with definite chronic kidney disease, demonstrated that the risk for low BMD remained significantly higher in subjects with low serum creatinine. Furthermore, one SD increase in serum creatinine was associated with a significant reduction in the occurrence of low BMD at TH and LS in both men and postmenopausal women. Those results were valid even after adjusting for confounding factors. Thus, serum creatinine could have a beneficial effect on BMD in subjects with normal renal function. The main pathogenesis for the deleterious effects of low serum creatinine on BMD could be that serum creatinine reflects one’s physical activity status as well as skeletal muscle mass, and both are important for maintaining bone health [26]. In addition, creatinine degradation is stimulated by reactive oxygen species and in particular by the hydroxyl radical. Fernández-Real et al. reported that telomere length of subcutaneous adipose tissue cells was positively associated with serum creatinine but not with GFR. In other words, decreased serum creatinine is associated with a marker of cellular senescence and oxidative stress and consequently decreased serum creatinine may result in deterioration of BMD via oxidative stress [27]. Finally, recent evidences suggest that insulin could stimulate osteoblast differentiation and have some anabolic properties for bone [28]. In our study, a higher serum insulin levels were detected in the upper normal creatinine group than in the low creatinine group. Therefore, lower serum creatinine group may have low potency of anabolic properties for bone due to increased clearance of insulin.

An interesting point of our study is that the relationship between serum creatinine and BMD was more prominent in men aged 45 years or older than in postmenopausal women. Although we could not elucidate why this association was more prominent in men than in women, it might be explained by the fact that females tend to have lower skeletal muscle mass than males. Additionally, increased body fat in postmenopausal women might alter the bone-muscle (creatinine) relationship. Conversion of androgens to estrogens in adipose tissue could have a modest effect on bone, especially in postmenopausal women [29]. However, in men, adipose tissue is not an important sex hormone source and as they have relatively small fat mass, it may not considerably influence on the bone-muscle (creatinine) relationship [30]. Those findings suggest that creatinine can differ by sex, and sex differences should be considered and weighed practically.

The major strength of this study is that the data were collected from a large nationwide survey that included 8,648 participants ages 45 to 95 years throughout Korea. This is the first observational study that extensively investigated the sex-specific association of low serum creatinine with BMD focusing on Korea’s general population. However, this study also has some limitations. First, given that serum creatinine is closely related to skeletal muscle mass, it could be influenced by other factors, such as drugs and other dietary variation. However, we could not collect all that information. Second, to define sarcopenia, we did not measure muscle strength or walk speed, as described by the European Working Group on Sarcopenia in Older People and the European Society for Clinical Nutrition and Metabolism special interest group [31]. Third, because this study used a cross-sectional design and not a longitudinal design, a causal relationship could not be definitively established. Fourth, we could not demonstrate whether this association between serum creatinine and BMD is independent of estimated glomerular filtration rate as there was collinearity in serum creatnine and estimated glomerular filtration rate. Finally, because all participants were single ethnic group, our results may not be representative of the other ethnic population.

In conclusion, our study is the largest population-based study to examine the sex-specific association between serum creatinine and BMD. An excessive decrease of serum creatinine was associated with greater deterioration of BMD in subjects with normal renal function, especially, in men. Our findings suggest that serum creatinine reflects an individual’s skeletal health status as well as muscle mass and that we can indirectly assess both bone and muscle health status from serum creatinine level, which can be easily measured. Monitoring serum creatinine could be helpful to the development of sex-specific strategies for the treatment and prevention of sarcopenia and low BMD.

## Supporting Information

S1 Dataset (ZIP)

S1 Table Partial correlations between serum creatinine and parameters. (DOCX)
