# Supplementary material for: Lower Serum Creatinine Is Associated with Low Bone Mineral Density in Subjects without Overt Nephropathy
Source: PLoS One. 2015 Jul 24;10(7):e0133062. doi: 10.1371/journal.pone.0133062 (PMC4514793; doi:10.1371/journal.pone.0133062)
Supplement: S1 Table — (DOCX) [file pone.0133062.s002.docx]

**S1 Table** Partial correlations between serum creatinine and parameters

| Variables | Serum creatinine (mg/dl) |
| --- | --- |
| Bone density (g/cm^2^) |  |
| Total hip BMD | 0.162^a^ |
| Femoral neck BMD | 0.221^a^ |
| Lumbar spine BMD | 0.274^a^ |
| Body composition |  |
| Body fat (%) | -0.309 ^a^ |
| Total skeletal muscle (kg) | 0.424^a^ |
| Appendicular skeletal muscle (kg) | 0.430^a^ |
| Appendicular skeletal muscle/(height)^2^ (kg/m^2^) | 0.362^a^ |

Values are partial correlation coefficients adjusted for age

^a^*P* < 0.001
